# Enhancing COVID-19 vaccination coverage using financial incentives: arguments to help health providers counterbalance erroneous claims

**DOI:** 10.4178/epih.e2021081

**Published:** 2021-10-06

**Authors:** Jelena Dotlic, Vida Jeremic Stojkovic, Paul Cummins, Marija Milic, Tatjana Gazibara

**Affiliations:** 1 Institute of Epidemiology, Faculty of Medicine, University of Belgrade, Belgrade, Serbia; 2 Department of Medical Education, Icahn School of Medicine at Mount Sinai, New York, NY USA; 3 Faculty of Medicine, University of Pristina temporarily settled in Kosovska Mitrovica, Kosovska Mitrovica, Serbia

**Keywords:** COVID-19, Vaccination, Reimbursement, Financial support

## Abstract

Financial reimbursements after receiving the coronavirus disease 2019 (COVID-19) vaccine have been criticized in the literature. This strategy has been described as payment to receive the vaccines, undue inducement, and unethical. We are aware that healthcare workers who work in primary healthcare, prevention, and public health may encounter similar reasons from people who refuse vaccination against COVID-19. For this reason, we are compelled to clarify these claims and provide arguments for all healthcare workers who might be challenged by such reasoning. In this critical review, we discuss why the claims against financial incentives that have been presented in the literature are erroneous.

For the past 18 months, the coronavirus disease 2019 (COVID-19) pandemic has been the main public health problem globally. Vaccines against COVID-19 became available 10 months ago; however, the coverage is not yet satisfactory. Vaccination against COVID-19 in Serbia began in December 2020 for all age groups [1]. Vaccination is voluntary and free of charge for all. Moreover, citizens are also offered the possibility to choose 1 of 4 vaccines (Pfizer-BioNtech, Sinopharm, Sputnik V, and Oxford-AstraZeneca). Serbian citizens can either make an appointment for vaccination through a web platform or telephone line set up just for this purpose or just walk in to a vaccination site without any appointment whatsoever to receive the vaccine. However, despite the relatively easy access and availability of the vaccine, it took a total of 8 months for 50% of people to receive at least 1 dose of a COVID-19 vaccine. The interest in vaccination was the highest in the first months after the beginning of systematic vaccination, while the increments in coverage have slowed down since May 2021.

In the context of global vaccine hesitancy, various strategies have been implemented in different countries to improve the coverage when the interest in vaccination began decreasing. Some countries organized lotteries, while others set up vaccination booths in major shopping malls and citizens were offered discounts for shopping in department stores and holiday packages after receiving the vaccine. Serbia was one of the first to allow all vaccinated citizens to register for a one-time offer of approximately 25 Euros in cash [2].

However, this strategy initiated a discussion, as it was characterized in some media reports and scientific literature as a payment to receive the vaccine, undue inducement, or even as an unethical approach [3]. We are aware that health systems worldwide may encounter similar allegations. These claims, therefore, must be clarified and arguments against them should be provided for all healthcare workers who work in primary healthcare, prevention, and public health in an effort to tackle the increasing vaccine hesitancy.

First, it is not accurate to claim that citizens are being paid to receive the vaccine. Like many other countries, Serbia established pandemic relief programs to offset financial losses sustained due to lockdown measures [4]. These programs are now just being expanded to provide additional financial support to citizens who have been vaccinated in the first 5 months of the COVID-19 vaccination program. Non-vaccinated people remain eligible for all other forms of pandemic relief except the additional 25 Euros. Payment is a reward offered for performing an activity; a substantial number, if not most, of the beneficiaries of this program will have qualified for this benefit before it was announced. Therefore, it is debatable that this is a payment that “changes the perception of the purpose of vaccination” [3]. This program is merely one of several government efforts to provide support during the COVID-19 crisis. Cash transfer programs could stimulate local business and commerce through the circulation of money. This could be a reason why other governments have also adopted similar strategies [5].

Second, calling these initiatives undue inducements commits a category error because an undue inducement, per Emanuel et al. [6], “requires substantial risk of serious physical, psychological, economic, or other harms, which threaten a person’s fundamental interests”. The idea is that an undue inducement would distort a potential research participant’s judgment to take an unacceptable risk for payment. This might be a considerable ethical problem in the context of human participation in clinical trials, which aim to test a new drug or procedure, and for which the prospect of benefit to participants is uncertain. However, COVID-19 vaccines have already been tested and authorized by regulatory agencies, which suggests that the risk is not substantial. In fact, they benefit the recipients [7].

Incentive programs are public health initiatives and should be assessed using the conceptual tools of public health ethics. Therefore, analyzing these initiatives using concepts from research ethics such as undue inducement and coercion is a mistake. This error is demonstrated in characterizing these incentives, which benefit people, as coercive. Coercion involves a threat to leave one worse off for refusing to comply with a request. These cash transfer offers involve no such threat, as the proposed incentive of 25 Euros corresponds to the daily wage of unqualified workers in Serbia, it is not an offer that is “too good to refuse.”

Third, applying a public health ethics lens to cash incentive programs does not provide support for the notion that these programs are ethically problematic. If we accept for the sake of argument that cash incentives do more strongly influence poor and disadvantaged people to accept vaccination, it is important to note that poor and disadvantaged people are indeed at greater risk of numerous health challenges including COVID-19 [8]. The benefits of vaccination to this population are expectedly high.

Like many European countries, Serbia has universal access to health care and vaccination is free of charge. Public health ethics frameworks endorse the values and principles of producing benefits while minimizing and fairly distributing burdens (e.g., restrictions on liberty), protecting disenfranchised communities, acting in a timely manner, and relying on evidence to inform practice [7]. Overall, we believe that a cash incentive program can comply with these values and principles. Around 60% of the total population registered to receive pandemic financial relief from the Serbian government in 2020/2021, and it is probable that the same proportion of the vaccinated population will register for additional financial benefits. It is unlikely that poor and disadvantaged Serbians are being asked to bear the burden of vaccine uptake. Moreover, because the anti-vaccination movement in Serbia is relatively strong, people feel free to refuse vaccination. However, there is a large population of people who are vaccine-hesitant and all strategies, including incentives, could potentially make them more interested in vaccination.

We agree that money could be spent on other means of encouraging vaccination uptake. In fact, different strategies are also being used to increase vaccination coverage in Serbia: participation of public figures and celebrities in vaccination; operating mobile vaccination sites across Serbia; and allowing foreign travel for vaccinated people without compulsory quarantine and costly testing for COVID-19.

## CONCLUSION

The vast majority of Serbians who would benefit from this incentive program received the COVID-19 vaccine before May 2021 and did so without any incentive in mind. In the face of enduring vaccine hesitancy and the absence of evidence that any of the initiatives to enhance vaccination coverage, including financial incentives, is superior to others, we believe it is appropriate for public health authorities to implement several options to help bring the end of the pandemic.

### Ethics statement

This paper is a perspective, so it did not need ethical approval.

## References

[1] World Health Organization Reginal Office for Europe (2021). Serbia’s COVID-19 vaccination campaign off to a strong start. https://www.euro.who.int/en/countries/serbia/news/news/2021/3/serbias-covid-19-vaccination-campaign-off-to-a-strong-start.

[2] Euronews with Agencies Serbia in ‘world first’ as citizens offered €25 to have COVID vaccine. https://www.euronews.com/2021/05/05/serbia-in-world-first-as-citizens-offered-25-to-have-covid-vaccine.

[3] Holt E (2021). Serbia begins paying citizens to receive a COVID-19 vaccine. Lancet.

[4] International Monetary Fund (2021). Policy responses to COVID-19. https://www.imf.org/en/Topics/imf-and-covid19/Policy-Responses-to-COVID-19.

[5] Wood P Maryland Gov. Hogan offers $100 to state employees who get COVID vaccine. https://www.baltimoresun.com/coronavirus/bs-md-vaccine-state-employees-20210503-4axlsdqnhzba7lylog3cplqml4-story.html.

[6] Emanuel EJ, Currie XE, Herman A, Project Phidisa (2005). Undue inducement in clinical research in developing countries: is it a worry?. Lancet.

[7] Centers for Disease Control and Prevention (2021). Benefits of getting a COVID-19 vaccine. https://www.cdc.gov/coronavirus/2019-ncov/vaccines/vaccine-benefits.html.

[8] Patel JA, Nielsen FB, Badiani AA, Assi S, Unadkat VA, Patel B (2020). Poverty, inequality and COVID-19: the forgotten vulnerable. Public Health.

[9] ten Have M, de Beaufort ID, Mackenbach JP, van der Heide A (2010). An overview of ethical frameworks in public health: can they be supportive in the evaluation of programs to prevent overweight?. BMC Public Health.

